# The Safety and Efficiency of Surgery with Colonic Stents in Left-Sided Malignant Colonic Obstruction: A Meta-Analysis

**DOI:** 10.1155/2014/407325

**Published:** 2014-05-14

**Authors:** Xiang Zhao, Bo Liu, Ende Zhao, Jiliang Wang, Ming Cai, Zefeng Xia, Qinghua Xia, Xiaoming Shuai, Kaixiong Tao, Guobin Wang, Kailin Cai

**Affiliations:** Gastrointestinal Surgery Department, Union Hospital, Tongji Medical College, Huazhong University of Science and Technology, Wuhan 430000, China

## Abstract

*Objective.* This meta-analysis is aimed at assessing the safety and efficiency of colonic self-expanding metallic stents (SEMS) used as a bridge to surgery in the management of left-sided malignant colonic obstruction (LMCO). *Methods.* A systematic search was conducted in PubMed, Web of Knowledge, OVID, Google Scholar, CNKI, and WANGFANG for relevant randomized trials comparing colonic stenting used as a bridge in semielective surgery versus emergency surgery from January 2001 to September 2013. *Result.* Five published studies were included in this systematic review, including 273 patients (140 male/133 female). 136 patients received semielective surgery after SEMS installation while 137 patients underwent emergency surgery without SEMS. SEMS intervention resulted in significantly lower overall colostomy rate (41.9% versus 56.2%, *P* = 0.02), surgical site infection rate (10.2% versus 19.7%, *P* = 0.03), and overall complication rate (29.2% versus 60.5%, *P* = 0.05). There was no statistic difference for the rate of primary anastomosis, anastomotic leak and operation-related mortality between two groups. *Conclusions.* semielective surgery with SEMS as a bridge for proper patients of LMCO can lower the overall rate for colostomy, surgical site infection, and complications.

## 1. Introduction

Emergency surgery was considered as the traditional treatment for left-sided malignant colonic obstruction (LMCO). However, the complication rate and mortality remained high for emergency surgery. Semielective radical surgery after preinstallation with self-expanding metallic stent (SEMS) to relieve colon obstruction showed promise for LMCO treatment. With SEMS application, surgeons gained more time for ameliorating patients' condition, bowel preparation, and preoperative assessment for tumor, which could improve the operative safety and efficiency by enhancing the rate of primary anastomosis while lowering the overall colostomy rate. It could raise the quality of life by avoiding mental and physical trouble caused by colostomy. Furthermore, it might decrease the mortality and overall complication rate due to improvement of patient condition and primary anastomosis rate.

In this paper, we further evaluated the safety and efficiency of SEMS as a bridge in LMCO by meta-analysis of randomized trials of semielective surgery after SEMS versus emergency surgery.

## 2. Materials and Methods

### 2.1. Database Search

A systematic search was conducted in PubMed, Web of Knowledge, OVID, Google Scholar, CNKI, and WANGFANG for relevant randomized trials comparing colonic stenting used as a bridge in semielective surgery versus emergency surgery from January 2001 to September 2013. The search strategy employed the following mesh headings and keywords alone or in combination, “SEMS,” “stents,” “left-sided colorectal cancer,” “obstruction,” “left-sided malignant colorectal obstruction,” “self-expanding metallic stents as a bridge to surgery,” “emergency surgery,” and “semielective surgery.”

### 2.2. Literature Screening and Assessment

The literature screening and assessment was conducted by two professionals with the following strict criteria.

#### 2.2.1. Inclusion Criteria

Studies were included in the present analysis when the following criteria were met:

 study about randomized controlled trials,
 study regarding patients with left-sided malignant colonic obstruction,
 study comparing semielective surgery with SEMS versus traditional emergency surgery.

Main statistical parameters include primary anastomosis rate, overall colostomy rate, anastomotic leak rate, overall complication rate, postoperative mortality within 30 days, and rate of surgical site infection.

#### 2.2.2. Exclusion Criteria

They are as follows:

 study about nonrandomized controlled trials,
 study about palliative treatment with SEMS,
 study about left colorectal obstruction caused by metastatic colorectal cancer,
 study about nonleft colorectal obstruction.

#### 2.2.3. Data Assessment

The data was assessed by one professional reviewer based on the criteria and further confirmed by another professional reviewer.

### 2.3. Statistical Analysis

Data analysis was performed with RevMan 5.0 provided by Cochrane. Risk ratio (RR) was used as statistical variable. Each effect variable was presented with 95% confidence interval (CI) and *P* = 0.05 was considered as statistical significance. *χ* ^2^ test and *I* ^2^ were employed to assess the heterogeneity. Mantel-Haenszel fixed effect model was used for data analysis if nonsignificant heterogeneity was detected (*P* > 0.1 and *I* ^2^ < 0.5). In case of significant heterogeneity (*P* < 0.1 or *I* ^2^ > 0.5) D-L random effect model was used for analysis.

## 3. Result

### 3.1. Literature Inclusion

**Figure 1:**
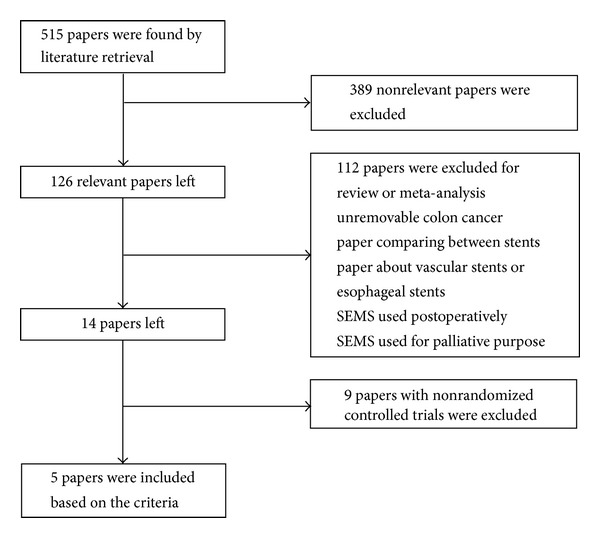
Flowchart for literature screening.

Total of five randomized controlled trials were included for meta-analysis [1–5] and the screening flowchart was shown in Figure 1. All were high-qualified literatures as evaluated with bias risk criteria recommended by Cochrane and Jadad scale [6] (Table 1). 273 patients (140 male/133 female) were included (Table 2) with 136 patients receiving semielective operation after SEMS and the other 137 patients receiving emergency surgery. Result was analyzed following the principle of intentional analysis.

### 3.2. Primary Anastomosis Rate

**Figure 2:**
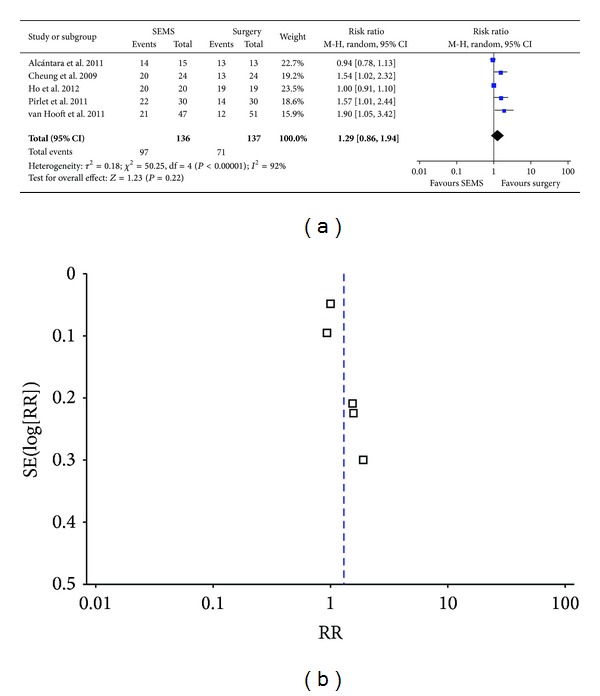
Primary anastomotic rate of semielective surgery after SEMS installation versus emergency surgery.

Random effects model was employed for the rate of primary anastomosis in the five papers as preanalysis showed heterogeneity (*P* < 0.00001, *I* ^2^ = 92%). The result displayed no statistical significance for primary anastomosis rate between SEMS and emergency surgery groups (RR 1.29; 95% CI, 0.86–1.94; *P* = 0.22), although SEMS group showed higher rate (71.3% versus 51.8%) (Figure 2(a)). And no bias was found by funnel plot (Figure 2(b)) and Egger's test (Table 3). Sensitivity analysis also indicated stable result (Table 4). Meta-analysis showed that 99% heterogeneity could be interpreted with sample size (*Z* = 3.0548, *P* = 0.0023, Table 5). All patients in both groups received primary anastomosis in the report by Ho and colleagues [3]. 12 out of 19 patients in emergency surgery group were treated with primary anastomosis after intestinal lavage during operation while 4 patients received primary anastomosis after intestinal lavage due to failure of stent installation. In the research by Alcántara et al., all patients in emergency surgery group received primary anastomosis after intestinal lavage during operation, and one patient was given Hartman operation due to peri-stent cellulitis [1]. The result showed homogeneity after we excluded the above-mentioned reports (*P* = 0.82; *I* ^2^ = 0%). Thus we performed fixed effect model analysis and found that the rate of primary anastomosis in SEMS group was significantly higher than that in emergency surgery group (62.4% versus 37.1%; RR 1.66; 95% CI 1.26–2.19; *P* = 0.0004).

### 3.3. Overall Colostomy Rate

**Figure 3:**
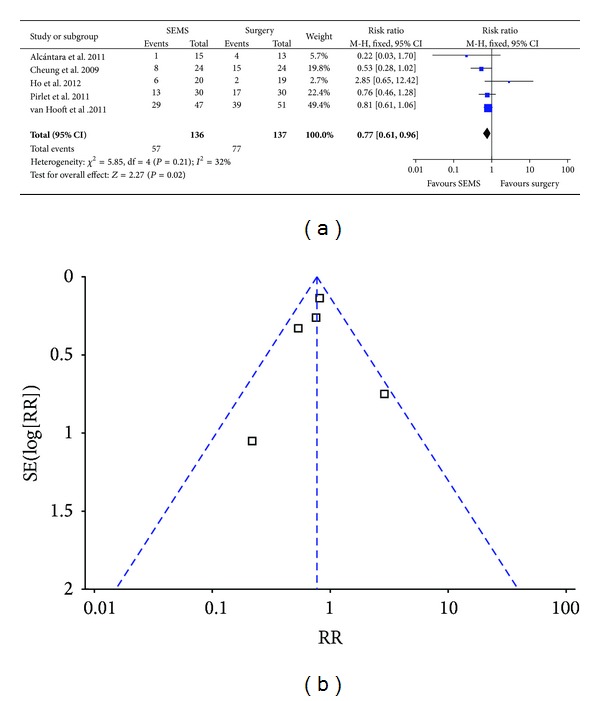
Forest plot and funnel plot of overall colostomy rate.

Fixed effect analysis model was employed since nonsignificant heterogeneity (*P* = 0.21, *I* ^2^ = 32%) and the result showed the overall rate of colostomy in SEMS group was significantly lower than that in emergency surgery group (41.9% versus 56.2%; RR 0.77; 95% CI 0.61–0.96; *P* = 0.02) (Figure 3(a)). No bias was found as indicated by funnel plot (Figure 3(b)) and Egger's test (Table 3).

### 3.4. Rate of Anastomotic Leak

**Figure 4:**
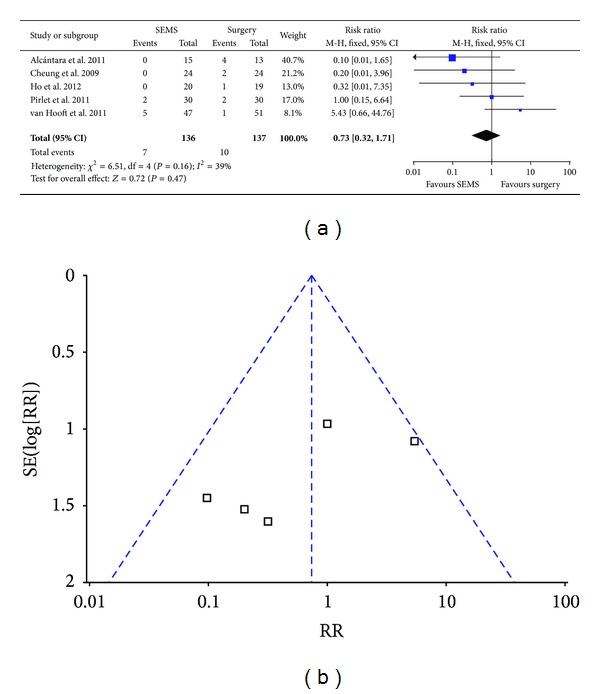
Forest plot and funnel plot of the rate of anastomotic leak.

Nonsignificant heterogeneity (*P* = 0.16, *I* ^2^ = 39%) was found by analysis. Therefore we analysed the data with fixed effect model and found that there was no significant difference for the rate of anastomotic leak between SEMS group and emergency surgery group (5.9% versus 6.6%; RR 0.73; 95% CI 0.32–1.71; *P* = 0.47) (Figure 4(a)). No bias was found by funnel plot (Figure 4(b)) and Egger's test (Table 3).

### 3.5. Overall Rate of Complication

**Figure 5:**
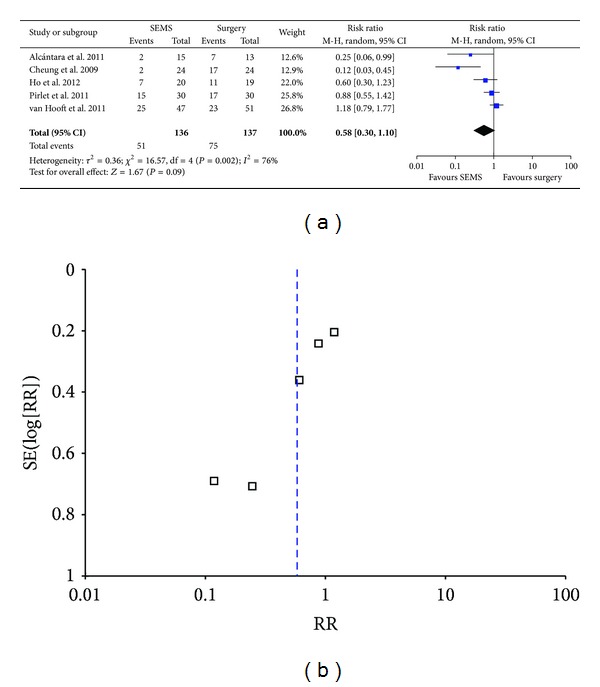
Forest plot and funnel plot of overall complication rate.

We used random effect model for analyzing overall rate of complication because of the heterogeneity among researches (*P* = 0.002, *I* ^2^ = 76%). The analysis indicated SEMS group had lower overall rate of complication than emergency surgery group without statistical significance (37.5% versus 54.7%; RR 0.58; 95% CI 0.30–1.10; *P* = 0.09) (Figure 5(a)). Furthermore, publication bias was found by funnel plot (Figure 5(b)) and Egger's test (Table 3). The result was not stable by metasensitivity analysis. However, it indicated statistical significance after removing the research by van Hooft et al. (Table 6). The authors considered that rate of complication may be overestimated due to more patients with complete intestinal obstruction (70%) and strict follow-up in van Hooft's research, which was distinct with others' reports. After excluding van Hooft et al.'s report, we found SEMS group had significantly lower overall rate of complication than emergency surgery group (29.2% versus 60.5%; RR 1.147; 95% CI 0.896–1.468; *P* = 0.05). Metaregression analysis indicated 83.9% heterogeneity may be interpreted by year of publication (*Z* = 2.4884, *P* = 0.0128, Table 7).

### 3.6. Postoperative Mortality within 30 Days

**Figure 6:**
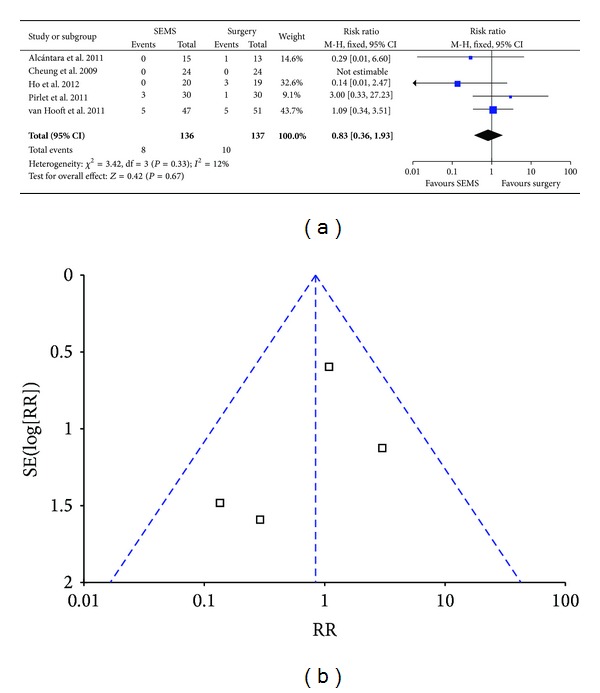
Forest plot and funnel plot of postoperative mortality within 30 days.

We employed fixed effect model for analysis since there was no heterogeneity (*P* = 0.33, *I* ^2^ = 12%) and found postoperative mortality with 30 days in SEMS group was slightly lower than that in emergency surgery group (5.9% versus 7.3%; RR 0.83; 95% CI 0.36–1.93; *P* = 0.67) (Figure 6(a)). Funnel plot (Figure 6(b)) and Egger's test (Table 3) indicated there was no bias for the analysis.

### 3.7. Rate of Surgical Site Infection (SSI)

**Figure 7:**
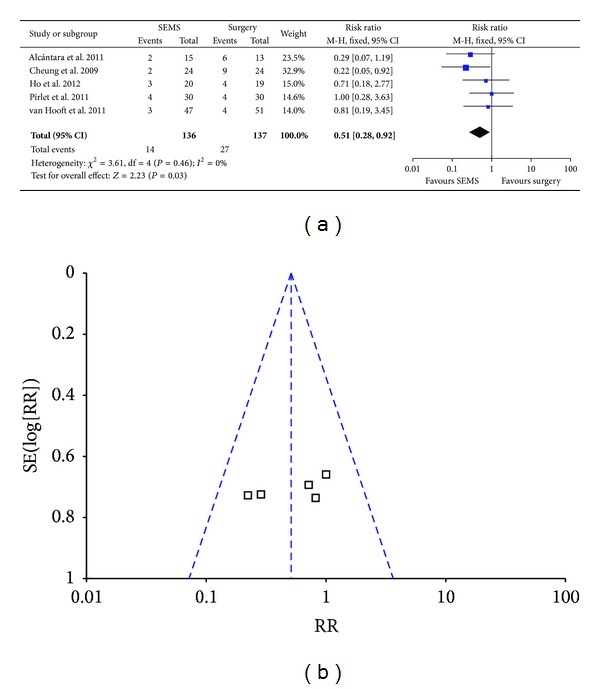
Forest plot and funnel plot of SSI.

Fixed effect model was used for analysis since there was no heterogeneity (*P* = 0.46, *I* ^2^ = 0) and the result showed the SSI rate in SEMS groups was significantly lower than that in emergency surgery group (10.2% versus 19.7%; RR 0.51; 95% CI 0.28–0.92; *P* = 0.03) (Figure 7(a)). No bias was found by funnel plot (Figure 7(b)) and Egger's test (Table 3).

### 3.8. Rate of Permanent Colostomy

The rate of permanent colostomy in SEMS group was lower than that in emergency surgery group (28.7% versus 38.7%; RR 0.77; 95% CI 0.57–1.04; *P* = 0.09).

## 4. Discussion

Tumor resection and proximal colostomy followed by stoma reversal to restore intestinal continuity is the most common surgery for left-sided malignant colonic obstruction because of the low rate of primary anastomosis under emergency condition. However, current treatment is considered too complicated with poor life quality and up to 40% complication rate and only 60% of patients received stoma reversal surgery [7, 8]. Therefore, more and more surgeons started to explore safer and more efficient operations for LMCO. Dohmoto reported 19 cases of nonresectable or metastatic rectal cancer with obstruction with laser recanalization or SEMS installation to relieve obstruction in 1991 [9]. Tejero et al. described the preliminary experience about the transition with SEMS installation to relieve obstruction for later decisive surgery with two colon cancer patients in 1993 [10]. Surgeons gained more time with SEMS application for ameliorating patients' condition, bowel preparation, and preoperative assessment for tumor stage, which could improve the operative safety and efficiency.

Watt et al. and Zhang et al. compared the clinical effect between surgery after SEMS installation and emergency surgery for colon cancer by review and meta-analysis, respectively [11, 12]. They found significant higher primary anastomosis rate and lower complication rate for SEMS group compared with emergency surgery group. No statistical significance was found for permanent rate of colostomy and postoperative mortality within 30 days although they were lower in SEMS group. However, the meta-analysis by Zhang et al. analyzed 6 retrospective studies and 2 randomized controlled studies. Similarly, more retrospective researches were included in the meta-analysis by de Ceglie et al. [13]. Therefore, potential inevitable bias may exist since there was obvious heterogeneity among the analysis about rate of anastomotic leak, postoperative mortality within 30 days, and long-term survival.

In this report, we focused on five randomized controlled trials and found that semielective surgery after SEMS installation had significant advantage over emergency surgery for the rate of overall colostomy, SSI, postoperative complication, and primary anastomosis. Semielective surgery after SEMS installation could improve patients' life quality and promote recovery by enhancing the rate of primary anastomosis and decreasing the rate of colostomy, postoperative complication, and SSI.

Although no statistical significance was detected, the difference between semielective surgery after SEMS and emergency surgery indicated SEMS application might have advantage for primary anastomotic rate (71.3% versus 51.8%), rate of anastomotic leak (5.9% versus 6.6%), and postoperative mortality within 30 days (5.9% versus 7.3%). However, apart from the above-mentioned advantage, the following issues should be emphasized regarding the transition of SEMS application in later decisive operation for LMCO.

First, stent installation may result in distinct clinical outcomes due to technical difficulty, such as abdominal infection and tumor implantation caused by stent-related perforation. Therefore, the ability of handling stent installation should be taken into consideration as the success rate and complication rate related to stent installation noticeably affect overall complication rate in SEMS group. Regarding the success rate of colonic stent application for transition of semielective surgery, one collecting analysis with 1198 patients in 54 studies revealed 92% technical success rate, 71.7% clinical success rate, and 3.76% perforation rate [14]. In comparison, the rate of successful stent installation and stent-related perforation was 70.2% (33/47), 12.8% (6/47), respectively, in van Hooft et al.'s report [5] and 46.7% (14/30), 6.7% (2/30), respectively, in Pirlet et al.'s report [4], leading to termination of the research ahead of schedule. Ho et al. employed subgroup analysis by excluding 6 cases that failed in stent installation and found SEMS group exhibited lower rate of colostomy and postoperative complication and faster intestinal recovery. Therefore, we speculated that SEMS application might achieve more advantages over surgery alone with optimal success rate.

Second, it is worth emphasizing the effect of stent application on long-term prognosis of tumor. Only one prospective randomized controlled study by Alcántara et al. analyzed the long-term mortality between semielective surgery after SEMS and emergency surgery [1]. Their result indicated there was no statistical significance for overall survival rate between two groups (*P* = 0.843). The disease-free interval for semielective surgery after SEMS and emergency surgery was 25.49 and 27.06 months, respectively (*P* = 0.096), with slightly higher recurrence rate for patients with SEMS (8 cases versus 2 cases, *P* = 0.055). In line with it, no significant difference was detected between two groups for long-term prognosis (survival rate of 1, 2, 3, and 5 years) in the systemic review by Watt et al. and meta-analysis by Zhang et al. [11, 12].

However, since the survival or recurrence was not considered as an end point in the five randomized trials analyzed in this study, the oncological safety cannot be assessed due to the lack of data. In fact, one retrospective controlled study challenged the safety of SEMS application as transition of later decisive operation for LMCO [15]. The result indicated the 5-year survival rate (25% versus 62%; *P* = 0.0003) and 5-year tumor-free survival rate (21% versus 48%; *P* = 0.02) in SEMS group were significantly lower than those in emergency surgery group, although semielective surgery after SEMS achieved more dissected lymph nodes and higher postoperative chemotherapy rate. Even after excluding cases with perforation and metastasis, significant difference was still detected for overall survival rate (*P* = 0.003) and 5-year survival rate (30% versus 67%; *P* = 0.001) between SEMS group and emergency surgery group [15]. Although the data from this retrospective study obviously differed from other reports, it strongly indicated that further research remained to be conducted for the effect of stent application on parameters such as long-term prognosis and life quality. Furthermore, chemotherapy before surgery for locally advanced colon cancer might be carefully considered to reduce the recurrence rate, which has the potential advantage in eradicating distant metastases and reducing the risk of incomplete surgical excision and the risk of tumor cell shedding during surgery by shrinking the primary tumor before surgery. Actually there are two clinical trials (FOxTROT and ECKINOXE) in progress to assess the effect of chemotherapy before surgery for locally advanced colon cancer. Following the clinical trials, we might propose a new therapeutic strategy for locally advanced colon cancer with the following 3 steps. First, create a stoma or apply the colonic stent to relieve the colonic obstruction. Second, apply chemotherapy by administrating neoadjuvant chemo to shrink primary tumor size and eradicate distant metastases. Third, perform surgery to resect the tumor tissue.

Taken together, the application of stent installation in operation for LMCO patients could enhance primary anastomotic rate while lowering the rate of colostomy, SSI, and postoperative complications. Although certain technical difficulty and risk accompanied with SEMS installation by colonoscopy under the condition of acute colonic obstruction, postoperative mortality remained comparable. Considering that all the included reports in this meta-analysis were prospective randomized controlled studies with limited amount, our conclusion remained to be further confirmed by more randomized controlled studies and strict long-term follow-up research.

## Figures and Tables

**Table:** (a) Bias risk profile

Author	Randomized trial (selection bias)	Allocation concealment (selection bias)	Blind selection (performance bias)	Blind evaluation (measurement bias)	Data deficient (attrition bias)	Literature quality
Alcántara et al. [1]	**−**	**−**	o	+	**−**	High
Cheung et al. [2]	**−**	**−**	o	+	**−**	High
Ho et al. [3]	**−**	**−**	o	o	**−**	High
Pirlet et al. [4]	**−**	**−**	o	+	**−**	High
van Hooft et al. [5]	**−**	**−**	o	o	**−**	High

Note: +: high risk, o: not available, and **−**: low risk.

**Table:** (b) Score of Jadad scale

Author	Generation of random sequence	Randomization concealment	Double-blind performance	Exit and attrition	Total
Alcántara et al. [1]	2	1	0	1	4
Cheung et al. [2]	2	1	0	1	4
Ho et al. [3]	2	1	0	1	4
Pirlet et al. [4]	2	1	0	1	4
van Hooft et al. [5]	2	1	0	1	4

Note: 1–3: low quality; 4–7: high quality.

**Table:** (a)

Author	Country	Year	Research type	Sample size	Age		Gender (number)		Group (number)	
					SEMS	Emergency surgery	Male	Female	SEMS	Emergency surgery
Cheung et al. [2]	China	2009	Randomized control	48	64.5 (39–68)	68.5 (27–86)	26	22	24	24
van Hooft et al. [5]	Netherlands	2011	Randomized control	98	70.4 (11.9)	71.4 (9.7)	51	47	47	51
Pirlet et al. [4]	France	2011	Randomized control	60	70.4 (10.3)	74.7 (30)	29	31	30	30
Alcántara et al. [1]	Spain	2011	Randomized control	28	71.9 (8.96)	71.2 (9.0)	12	16	15	13
Ho et al. [3]	Singapore	2012	Randomized control	39	68 (51–85)	65 (49–84)	22	17	20	19

**Table:** (b)

Author	Number and success rate of stent installation	Surgical procedure	Primary anastomotic rate		Rate of anastomotic leak	
			SEMS	Emergency	SEMS	Emergency
Cheung et al. [2]	20 (83.0)	Semielective laparoscopic surgery after SEMS versus emergency laparotomy	83.3%	54%	0%	8.3%
van Hooft et al. [5]	33 (70.0)	Semielective laparotomy after SEMS versus emergency laparotomy	44.7%	23.5%	10.7%	1.9%
Pirlet et al. [4]	14 (47.0)	Semielective laparotomy after SEMS versus emergency laparotomy	73.3%	46.7%	6.7%	6.7%
Alcántara et al. [1]	15 (100.0)	Semielective one-stage laparotomy after SEMS versus emergency one-stage laparotomy with intestinal lavage	93.3%	100%	0%	30.8%
Ho et al. [3]	14 (70.0)	Semielective laparotomy or laparoscopy after SEMS versus emergency laparotomy	100%	100%	5.0%	0%

**Table:** (c)

Author	Overall colostomy rate		Permanent colostomy rate		Overall rate of complication		Postoperative mortality within 30 days		SSI		Rate of pulmonary infection	
	SEMS	Emergency	SEMS	Emergency	SEMS	Emergency	SEMS	Emergency	SEMS	Emergency	SEMS	Emergency
Cheung et al. [2]	33.3%	62.5%	0%	25.0%	8.3%	70.8%	0%	0%	8.3%	37.5%	0%	4.2%
van Hooft et al. [5]	61.7%	76.5%	57.5%	66.7%	53.2%	45.1%	10.7%	9.8%	6.4%	7.9%	6.4%	2.0%
Pirlet et al. [4]	43.3%	56.7%	30.0%	26.7%	50.0%	56.7%	10.0%	3.3%	13.3%	13.3%	3.3%	10%
Alcántara et al. [1]	6.7%	30.8%	6.7%	30.8%	13.3%	53.9%	0%	7.7%	13.3%	46.2%	0%	0%
Ho et al. [3]	30.0%	10.5%	10.0%	5.3%	35.0%	57.9%	0%	15.8%	15.0%	21.1%	10%	10.5%

**Table 3:** Result of meta-analysis.

Content	Sample size	Model of meta-analysis	RR (95% CI)	Heterogeneity test		*P* value of Egger's test
				*P* value	*I* ^2^ (%)	
Primary anastomotic rate	273	Random effect model	1.29 (0.86–1.94)	0.00001	92	0.0503
Overall colostomy rate	273	Fixed effect model	0.77 (0.61–0.96)	0.2132	32	0.8785
Rate of anastomotic leak	273	Fixed effect model	0.73 (0.32–1.71)	0.1639	39	0.1648
Overall complication rate	273	Random effect model	0.58 (0.30–1.10)	0.00276	76	0.0065
Postoperative mortality within 30 days	273	Fixed effect model	0.83 (0.36–1.93)	0.3312	12	0.4911
SSI	273	Fixed effect model	0.51 (0.28–0.92)	0.460	0	0.276

**Table 4:** Sensitivity test for meta-analysis of primary anastomotic rate of semielective surgery after SEMS installation versus emergency surgery.

Excluded study	(95% CI) After exclusion	Heterogeneity test	
		*P* value	*I* ^²^ (%)
Alcántara et al. 2011 [1]	1.359 (0.995, 1.857)	0.01	68.045
Cheung et al. 2009 [2]	1.179 (0.879, 1.583)	0.031	82.419
Ho et al. 2012 [3]	1.354 (0.967, 1.895)	0.011	68.003
Pirlet et al. 2011 [4]	1.176 (0.882, 1.567)	0.029	82.065
van Hooft et al. 2011 [5]	1.147 (0.896, 1.468)	0.037	77.705

**Table 5:** Metaregression analysis of primary anastomotic rate of semielective surgery after SEMS installation versus emergency surgery.

Variable	*Z* value	*P* value	Tau^2^ value
Gender	0.2745	0.7837	0.0991
Publication year	−0.7392	0.4598	0.0632
Sample size	3.0548	0.0023	0.0006
Age in SEMS group	−0.1638	0.8699	0.0903
Age in emergency group	0.1639	0.8698	0.0897

**Table 6:** Sensitivity test for meta-analysis of overall complication rate of semi-elective surgery after SEMS installation versus emergency surgery.

Excluded study	RR (95% CI) after exclusion	Heterogeneity test	
		*P* value	*I* ^²^ (%)
Alcántara et al. 2011 [1]	0.625 (0.283, 1.383)	0.009	84.667
Cheung et al. 2009 [2]	0.817 (0.534, 1.249)	0.099	47.298
Ho et al. 2012 [3]	0.495 (0.178, 1.379)	0.003	88.043
Pirlet et al. 2011 [4]	0.446 (0.166, 1.198)	0.002	81.895
van Hooft et al. 2011 [5]	0.424 (0.181, 0.995)	0.022	73.792

**Table 7:** Metaregression analysis of overall complication rate of semielective surgery after SEMS installation versus emergency surgery.

Variable	*Z* value	*P* value	Tau^2^ value
Gender	0.0497	0.9603	0.8032
Publication year	2.4884	0.0128	0.0839
Sample size	1.6821	0.0925	0.2822
Age in SEMS group	1.4836	0.1379	0.2976
Age in emergency group	1.6183	0.1056	0.3406

## References

[1] Alcántara M, Serra-Aracil X, Falcó J, Mora L, Bombardó J, Navarro S (2011). Prospective, controlled, randomized study of intraoperative colonic lavage versus stent placement in obstructive left-sided colonic cancer. *World Journal of Surgery*.

[2] Cheung HYS, Chung CC, Tsang WWC, Wong JCH, Yau KKK, Li MKW (2009). Endolaparoscopic approach vs conventional open surgery in the treatment of obstructing left-sided colon cancer: a randomized controlled trial. *Archives of Surgery*.

[3] Ho K-S, Quah H-M, Lim J-F, Tang C-L, Eu K-W (2012). Endoscopic stenting and elective surgery versus emergency surgery for left-sided malignant colonic obstruction: a prospective randomized trial. *International Journal of Colorectal Disease*.

[4] Pirlet IA, Slim K, Kwiatkowski F, Michot F, Millat BL (2011). Emergency preoperative stenting versus surgery for acute left-sided malignant colonic obstruction: a multicenter randomized controlled trial. *Surgical Endoscopy and Other Interventional Techniques*.

[5] van Hooft JE, Bemelman WA, Oldenburg B (2011). Colonic stenting versus emergency surgery for acute left-sided malignant colonic obstruction: a multicentre randomised trial. *The Lancet Oncology*.

[6] Jadad AR, Moore RA, Carroll D (1996). Assessing the quality of reports of randomized clinical trials: is blinding necessary?. *Controlled Clinical Trials*.

[7] Pearce NW, Scott SD, Karran SJ (1992). Timing and method of reversal of Hartmann’s procedure. *British Journal of Surgery*.

[8] Desai DC, Brennan EJ, Reilly JF, Smink RD (1998). The utility of the Hartmann procedure. *The American Journal of Surgery*.

[9] Dohmoto M (1991). New method: endoscopic implantation of rectal stent in palliative treatment of malignant stenosis. *Endoscopia Digestiva*.

[10] Tejero E, Mainar A, Fernandez L, Tobio R, De Gregorio MA (1994). New procedure for the treatment of colorectal neoplastic obstructions. *Diseases of the Colon and Rectum*.

[11] Watt AM, Faragher IG, Griffin TT, Rieger NA, Maddern GJ (2007). Self-expanding metallic stents for relieving malignant colorectal obstruction: a systematic review. *Annals of Surgery*.

[12] Zhang Y, Shi J, Shi B, Song C-Y, Xie W-F, Chen Y-X (2012). Self-expanding metallic stent as a bridge to surgery versus emergency surgery for obstructive colorectal cancer: a meta-analysis. *Surgical Endoscopy and Other Interventional Techniques*.

[13] de Ceglie A, Filiberti R, Baron TH, Ceppi M, Conio M (2013). A meta-analysis of endoscopic stenting as bridge to surgery versus emergency surgery for left-sided colorectal cancer obstruction. *Critical Reviews in Oncology / Hematology*.

[14] Sebastian S, Johnston S, Geoghegan T, Torreggiani W, Buckley M (2004). Pooled analysis of the efficacy and safety of self-expanding metal stenting in malignant colorectal obstruction. *The American Journal of Gastroenterology*.

[15] Sabbagh C, Browet F, Diouf M (2013). Is stenting as, “a bridge to surgery” an oncologically safe strategy for the management of acute, left-sided, malignant, colonic obstruction? A comparative study with a propensity score analysis. *Annals of Surgery*.

